# A more than 20-year follow-up of pain and disability after anterior cervical decompression and fusion surgery for degenerative disc disease and comparisons between two surgical techniques

**DOI:** 10.1186/s12891-023-06503-w

**Published:** 2023-05-22

**Authors:** Anna Hermansen, Rune Hedlund, Peter Zsigmond, Anneli Peolsson

**Affiliations:** 1 Department of Health, Medicine and Caring Sciences, Unit of Physiotherapy, Linköping University, Building 511, Entrance 76, Level 15, Linköping, SE-581 83 Sweden; 2 Aleris Ortopedi, Nacka Specialistsjukhus, Stockholm, Sweden; 3 Department of Biomedical and Clinical Sciences, Linköping University, Linköping, Sweden and Department of Neurosurgery, Region Östergötland, Linköping, Sweden; 4 Department of Health, Medicine and Caring Sciences, Unit of Physiotherapy, Linköping, Sweden; Occupational and Environmental Medicine Centre and Department of Health, Medicine and Caring Sciences, Unit of Clinical Medicine, Linköping University, Linköping, Sweden

**Keywords:** Cloward procedure, Cervical intervertebral fusion cage, Long-term follow-up, Pain, Disability

## Abstract

**Background:**

Follow-ups more than 20 years after neck surgery are extremely rare. No previous randomized studies have investigated differences in pain and disability more than 20 years after ACDF surgery using different techniques. The purpose of this study was to describe pain and functioning more than 20 years after anterior cervical decompression and fusion surgery, and to compare outcomes between the Cloward Procedure and the carbon fiber fusion cage (CIFC).

**Methods:**

This study is a 20 to 24-year follow-up of a randomized controlled trial. Questionnaires were sent to 64 individuals, at least 20 years after ACDF due to cervical radiculopathy. Fifty individuals (mean age 69, 60% women, 55% CIFC) completed questionnaires. Mean time since surgery was 22.4 years (range 20,5–24). Primary outcomes were neck pain and neck disability index (NDI). Secondary outcomes were frequency and intensity of neck and arm pain, headache, dizziness, self-efficacy, health related quality of life or global outcome. Clinically relevant improvements were defined as 30 mm decrease in pain and a decrease in disability of 20 percentage units. Between-group differences over time were analyzed with mixed design ANOVA and relationships between main outcomes and psychosocial factors were analyzed by Spearman´s rho.

**Results:**

Neck pain and NDI score significantly improved over time (p  <  .001), with no group differences in primary or secondary outcomes. Eighty-eight per cent of participants experienced improvements or full recovery, 71% (pain) and 41% (NDI) had clinically relevant improvements. Pain and NDI were correlated with lower self-efficacy and quality of life.

**Conclusion:**

The results from this study do not support the idea that fusion technique affects long-term outcome of ACDF. Pain and disability improved substantially over time, irrespective of surgical technique. However, the majority of participants reported residual disability not to a negligible extent. Pain and disability were correlated to lower self-efficacy and quality of life.

## Background

Anterior Cervical Decompression and Fusion (ACDF), with or without cages or plates to support the segment, is a common surgical technique to treat cervical radiculopathy due to degenerative disc disease [1]. The use of a cage in ACDF has the theoretical advantage of restoring disc height and preventing graft collapse [2]. A Cochrane report concluded, however, that no added benefit of cages over autograft was seen [3]. A systematic review of a variety of surgical techniques showed an overall success rate of approximately 80% [1], and long-term follow-ups show success rates of 78–88% [4–8]. Long-term pain and disability outcomes also significantly improve on group level [4, 5, 7, 8]. However, in the present series of patients as well as in another study remaining disability was observed at 10-year follow-ups [7, 8]. Despite overall group improvements, some individuals report remaining, or recurrent, disability and pain at short [9] and long-term [5, 6, 10–12] after ACDF.

Degenerative processes of the cervical spine progress with age and may result in symptomatic adjacent segment disease [13], and may be accelerated after ACDF due to mechanical alterations after fusion [14]. Follow-ups 20 years after surgery are extremely rare [5, 6, 12], and often lack assessments of disability and psychosocial factors. No previous studies have investigated differences in pain and disability between the Cloward Procedure (CP) and a fusion cage more than 20 years after surgery. Headache and dizziness may be caused by impairments of the cervical spine and has previously been reported in patients after ACDF. However, no studies have evaluated this more than 20 years after surgery.

The purpose of this study was to compare pain and disability outcomes between Cloward procedure and a carbon fiber interbody fusion cage (CIFC) over time, to describe self-reported outcomes of pain, disability, and psychosocial factors more than 20 years after surgery, and to investigate associations between neck pain and disability, and self-efficacy and quality of life.

## Methods

### Study design

A 20-24-year follow-up of a randomized controlled trial comparing self-reported outcomes of neck pain and disability after CP and a CIFC [2].

An administrator at the neurosurgical clinic identified individuals from the original study [2] and still available for participation, and questionnaires were sent via the post. One-hundred and three consecutive patients were randomized to, and 95 were treated (1995–1998) with either the CIFC [2] or the Cloward Procedure [15]. See Table 1 for inclusion and exclusion criteria. Out of the original sample 64 eligible participants received an invitation to participate. An attending nurse performed the randomization by selecting a note marked with either “Cloward Procedure” or “CIFC” [2].

**Table 1:** Inclusion criteria of the original randomized controlled trial

Inclusion criteria	Exclusion criteria
• > 6 months of neck pain and radiculopathy of degenerative origin • MRI and clinical findings of cervical nerve root compression	• myelopathy • psychiatric disorder • drug abuse • previous spine surgery

MRI = magnetic resonance imaging

### Participants

Fifty individuals (30 women, 20 men, median age 69 years, range 52–83) completed the questionnaires 20.5 to 24 (mean 22.4) years after surgery (Table 2) and were included in this more than 20-year follow-up. Twenty-three individuals had been operated with Cloward Procedure and 27 with the CIFC. Fourteen individuals did not complete the questionnaire. (5 declined to respond due to other medical issues, 3 stated no reason for declining, and 6 did not respond despite several reminders); thus, 78% of the potential participants (53% of those initially operated on) answered the questionnaires.

Of the participants, 32 individuals had surgery at one cervical level, 16 at two levels, and 2 at three levels. Eight participants had at least one additional surgery during the follow-up period (not significant between groups). There was a similar distribution between the two surgical groups regarding age, gender, and preoperative pain and disability ratings. Two-year radiographs showed fused operated segment(s) in 33 individuals (missing data in 3 individuals), with a significant difference between groups (fusion rates: Cloward procedure = 90%, CIFC = 54%, p = .010) (Table 2).

**Table 2:** Background Data of Study Participants

	Total (n = 50–46)	CIFC	CP	Group differences
Type of surgery		27 (54)	23 (46)	
Gender n (%)				.393
Women	28 (56)	17 (63)	11 (48)	
Men	22 (44)	10 (37)	12 (52)	
Age, mean years (SD)	69 (8.4)	70 (8.5)	68 (8.3)	.382
Time since surgery, mean years (SD)	22.4 (0.9)	22.4 (0.9)	22.5 (0.9)	.651
No. of levels n (%)				.077
1	32 (64)	14 (52)	18 (78)	
2	16 (32)	12 (44)	4 (17)	
3	2 (4)	1 (4)	1 (5)	
Additional^#^ surgeries n (%)				.604
No	39 (81)	20 (77)	19 (86)	
Yes	7 (15)	4 (15)	3 (14)	
Yes, more than once	2 (4)	2 (8)	0 (0)	
Fusion status at 1 year n (%)				.010*
Fusion	33 (70)	14 (54)	19 (90)	
Non-fusion	14 (28)	12 (46)	2 (10)	
Baseline pain, mm VAS N = 49 (mean (SD))	69 (19.1)	69 (15.9)	70 (22.4)	.847
Baseline disability, NDI% N = 49 (mean (SD))	34 (8.6)	33 (9.0)	36 (8.0)	.258

CIFC = cervical intervertebral fusion cage, CP = Cloward procedure, NDI = neck disability index, VAS = visual analogue scale, * = significant differences, # additional surgeries includes both revision surgery (same segment) and additional surgery (different segment)

### Ethical considerations

This study was performed in accordance with the Declaration of Helsinki ethical principles for medical research and was approved by the Regional Ethics Review board in Linköping, Sweden (Dnr: M119-08, 2010/101 − 32, 2012/416 − 31, 2018/330 − 31). All participants received written information and provided written informed consent to participate prior to analyzing data. Data was anonymized and stored in a secure locker, and as encrypted computer files at Linköping University.

### Interventions

#### Surgical procedures

The Cloward procedure was performed according to standard techniques using bicortical iliac autograft to fill the empty disc space after removal of the disc and osteophytes [2]. The autograft was harvested through a 5-cm skin incision using a Cloward dowel cutter [15]. The CIFC surgical technique was performed in a similar way to the Smith-Robinson technique [16], with the addition of a carbon fiber cage to support the segment [2].

#### Postoperative care

Postoperatively, all patients used a Philadelphia collar for six weeks. Most participants received customary, not designed for the study, physiotherapy (information/advice from the physiotherapist at the Department of Spine Surgery). After removal of the collar, and if needed, patients were referred to a physiotherapist in primary healthcare.

### Data collection

Data was collected through self-reported questionnaires that were distributed via the post. In two patients, a short, structured interview was conducted by telephone to obtain ratings of primary outcomes and global outcome.

#### Primary outcome measures

Pain and disability were collected at baseline, 10 and 20-year follow-ups. Neck pain “right now” was assessed with the 100 mm Visual Analogue Scale (VAS) [17].

Neck-related disability was assessed with the Neck disability index (NDI) score (0 = no disability, 100 = complete disability).

#### Secondary outcome measures

Secondary outcomes were collected at the 20-year follow-up. Frequency of neck/arm pain and headache, dizziness and other neck-related problems were rated on a 5-point scale rating scale (0 = never, 4 = constant). For statistical reasons, ratings were categorized as never (0), occasionally [1], or daily/constantly [2–4].

Specific pain ratings of neck, arm, and headache were collected using the 100 mm VAS. Individuals with headache related to their neck completed the Swedish version of the HIT-6 questionnaire [18].

Dizziness at rest and during movement, and self-rated balance problems were assessed by a 100 mm VAS [19]. Participants with dizziness and/or perceived balance problems completed the 25 items Dizziness Handicap Inventory (DHI) [20, 21] to quantify the impact of dizziness on daily life (0 = no handicap to 100 = severe/maximal handicap).

A 6-point rating scale ranging from completely recovered to much worse [1–6] assessed global outcome (modified Odom). Symptom satisfaction was rated on a 7-point rating scale from 0 = happy to 6 = miserable [22]. The individual’s beliefs in their own ability to perform their daily activities despite pain was assessed with the Swedish version of the self-efficacy scale (0 = not at all confident to 200 = very confident) [23]. Health related quality of life was assessed using the EuroQol 5 dimensions (EQ-5D-3 L) index score (0 = poor overall health, 1 = good overall health) and the EuroQol 100 vertical VAS (0 = worst imaginable health, 100 = perfect health) [24].

### Statistical analyses

Demographic and cross-sectional descriptive data was analyzed using parametric or non-parametric tests depending on the measure and normality of data.

Differences between surgical groups and across three time periods (baseline, 10- and 20-year follow-ups) was analyzed using a 3 × 2 mixed design analysis of variance (ANOVA). Forty-five (VAS) and 42 (NDI) individuals completed ratings at all three time-points and were included in the ANOVA. Greenhouse-Geisser correction was used when the sphericity assumption was not met. Additional pairwise comparisons between time-points were performed if main effects were significant. Differences in improvements from baseline in VAS neck pain and NDI based on fusion status were analyzed with one-way analysis of covariance (ANCOVA), adjusted for baseline values.

A p-value of < .05 was considered statistically significant. Effect sizes were considered small if Cohen’s d was > 0.2, intermediate if > 0.5, and large if > 0.8. Global outcome ratings of 1 (completely recovered) to 3 (better) were categorized as improved, whereas 4 (unchanged) to 6 (much worse) was categorizes as not improved. A 30 mm reduction in VAS and a change score of 20% units in the NDI were used as cut-offs to determine clinically relevant improvements (CRI) in pain intensity [25] and disability [26].

Spearman’s rho was used to evaluate the relationship between pain and disability, and psychosocial factors.

## Results

Descriptive data on symptoms, disability and other health outcomes are presented in Table 3.

**Table 3:** Descriptive data of primary and secondary data at 20 years after surgery

Outcome Measure	Total	CP (n = 23–13)	CIFC (n = 27 − 20)	p-values
Neck pain intensity n = 50 (VAS, median (Q1–Q3))	8.5 (2–37)	5.0 (1–35)	10.0 (3–43)	.329
(VAS mean (SD))	19.7(23.5)	18.2 (26.1)	21.0 (21.5)	.677
Neck pain frequency n = 47				.846
Never	6 (13)	2	4	
Occasionally	26 (55)	13	13	
Daily/constantly	15 (32)	7	8	
Arm pain intensity n = 50 (VAS, median (Q1–Q3))	9 (1–25)	9 (1–33)	13 (1–25)	.969
Arm pain frequency n = 46				.374
Never	20 (43)	12	8	
Occasionally	9 (20)	3	6	
Daily/constantly	17 (37)	7	10	
NDI n = 47 (mean (SD))	22.6 (18.2)	21.6 (18.3)	23.4 (18.4)	.729
Headache intensity n = 48 (VAS, median (Q1–Q3))	3 (0–16)	2.5 (0.8)	9.5 (17.3)	.967
Headache frequency n = 46				.652
Never	14 (30.5)	8	6	
Occasionally	20 (43.5)	8	12	
Daily/constantly	12 (26)	6	6	
Headache impact test n = 32 (mean (SD))	49.5 (12.7)	47.9 (15.8)	50.8 (9.4)	.529
Dizziness intensity, rest n = 45 (VAS, median (Q1–Q3))	2 (0–18)	3 (0.5–19.5)	2 (0.0–18.8)	.836
Dizziness intensity, movement n = 45 (VAS, median(Q1–Q3))	5 (2–24)	5 (2–21)	8.5 (1–45)	.918
Balance problems intensity n = 46 (VAS, median (Q1–Q3))	18 (3–42)	13 (2.8–48.5)	21 (2.8–48.5)	.440
Dizziness/unsteadiness frequency n = 46				.707
Never	16 (35)	9 (40.9)	7 (29)	
Occasionally	15 (32.5)	7 (31.8)	8 (33)	
Daily/constantly	15 (32.5)	6 (27.3)	9 (38)	
Impact of dizziness n = 33 (DHI, mean (SD))	34.3 (21)	35.4 (25.1)	33.6 (18.8)	.817
Symptom satisfaction n = 47				.926
Delighted/Pleased	23 (49)	11 (52)	12 (46)	
Mostly satisfied	9 (19)	4 (19)	5 (19)	
Mostly dissatisfied/Unhappy	15 (32)	6 (29)	9 (35)	
Self-efficacy n = 47 (mean (SD))	148.5 (40.0)	150.0 (43.8)	147.4 (37.6)	.838
HRQoL (EQ5D) n = 46 (mean (SD))	.743 (.227)	.759 (.202)	.729 (.249)	.661
Health status (EQ VAS) n = 47 mean (SD)	75 (20.2)	75 (20.4)	76 (20.4)	.908
Global outcome^#^ (n = 50)				.307
Much improved	26 (52)	13 (57)	13 (48)	
Improved	18 (36)	6 (26)	12 (44)	
Unchanged/worse	6 (12)	4 (17)	2 (8)	
CRI in neck pain (n (%))				.528
Improved	35 (71)	15 (65)	20 (77)	
Not improved	14 (29)	8 (35)	6 (23)	
CRI in disability (n (%))				.370
Improved	19 (41)	11 (50)	8 (33)	
Not improved	27 (59)	11 (50)	16 (67)	

VAS = 100 mm visual analogue scale, NDI = neck disability index, DHI = dizziness handicap inventory, HRQoL = health-related quality of life, Eq. 5D = EuroQol 5 dimensions, EQ VAS = EuroQol 100 vertical VAS, CRI = clinically relevant improvement, ^#^modified Odom

### Differences within and between groups in pain and disability

There were no differences over time between surgical groups in pain (p = .951) or disability (p = .688). There was a significant main effect over time in pain F(1.59, 68.41) = 78.92, p < .001, Cohen’s d > 1.0. Post hoc analyses showed significant improvements from baseline to 10 years as well as from 10 to 20 years after surgery in both groups (CP, p < .001 – .007, CIFC, p < .001 – .013). There was a significant main effect in disability over time F(2,80) = 9.30, p < .001, Cohen’s d = 0.96. Post-hoc analyses showed significant improvements from baseline to 20-year follow-up in the CP group (p = .014), but not in the CIFC group (p = .066). Analyses showed no improvements from 10 to 20 years in either group (CP, p = .41, CIFC, p = 1.00).

Descriptive data on pain divided by fusion status at 2-year follow-up show that non-fused individuals had smaller reductions in pain over time than fused individuals. These reductions were not significantly different (p = .052) between groups based on fusion status. In NDI, the 2 individuals who had a non-fused CP surgery differed from the other by rating increased disability at 20-year follow-up. Analyses showed that individuals with a documented fusion (independent of type of surgery) had significantly larger improvements than those with non-fusion (mean difference: 13.4, 95% CI 1.3–25.6, p. = .031, Cohen’s d = 0.714) when adjusted for baseline NDI values.

### Self-reported improvements at 20-year follow-up

Of the 50 participants, 44 (88%) (Table 3) rated their problems as improved according to the global rating of change scale. Nineteen (83%) individuals in CP group and 25 (92%) in the CIFC group were improved (p = .307). Thirty-five individuals (71%) had clinically relevant improvements pain, (15 CP, 20 CIFC, p = .528), and 19 individuals (41%) in disability from preoperatively to 20-year follow-up (11 CP, 8 CIFC, p = .370) (Table 3).

### Differences in secondary outcomes

There were no differences between individuals based on surgical groups in any secondary measures of symptoms, disability, or other outcomes (p = .307 – .969). Most individuals experienced neck and arm pain at least occasionally (87% and 57% respectively), but median neck and arm pain were both 9 mm VAS (Table 3). Headache was experienced at least occasionally by 69% of participants, but with low intensity score (median 3 mm VAS). Mean disability score was 22.6 (SD 18.2) (Table 3). Dizziness was rated as 2 and 5 mm VAS, however median balance problems was 18 mm and mean disability due to dizziness was 34 on the DHI (Table 3). Mean health status was 75 (SD 20.2). Overall, 68% of participants were pleased or mostly satisfied with the outcome (Table 3).

### Associations between pain and disability scores, and psychosocial outcomes

Self-efficacy was correlated to the 20-year outcome of neck pain (rho = − 0.483, p = .001) and NDI (rho = − 0.619, p < .001). Quality of life was also significantly correlated to neck pain (rho = − 0.383, p = .008) and NDI (rho =-0.552 and p < .001).

## Discussion

The most important results from this study were that no differences were present between CP or CIFC at this unique ultra-long-term follow-up. Both pain and disability remained improved during this 20-year period. Also 71% of participants had a CRI in pain compared to 41% in disability, and 88% were improved according to in global outcome ratings (modified Odom). These main results are in line with previous follow-ups at 2 years [2], 6 years [27], and 10 years [8] years after surgery.

Notably, pain ratings continued to improve even after the 10-year follow-up, but NDI did not. This might be due to participants with pain who continue to live their lives in a somewhat limited way, thus reducing the mechanical strain on the neck, but with a consequent impact on daily life. This has been described by individuals 2 years after surgery [28] and may apply to other individuals as well. Most participants were also retired from work, possibly reducing the strain on the neck, and subsequently their pain ratings. There was no worsening in pain or NDI, a result that may be surprising considering the accelerating degenerative process that occurs with age, and possible adjacent segment degeneration after surgery [13, 14], which have been reported at 20-year follow-ups at 61–96% [5, 12]. This follow-up did not include radiographic evaluation, which would have allowed an assessment of the possible impact of degenerative changes. A few participants had however had additional surgeries which might have addressed such changes and thus altered the group means to the better. The lack of radiographic assessment was due to logistic reasons, with participants spread over different parts of Sweden.

Global outcome (modified Odom) according to the dichotomized scores was higher than at the 2- and 10- year follow-ups [2, 8], and approximately the same as at another 20-year follow-up that evaluated patient reported overall improvement [6]. In the present study, CRI in pain was 71% and CRI in disability was 41%. These rates are higher than at 10-year follow-up; however, they are lower than the rates of global outcome (modified Odom). Higher rates compared to earlier follow-ups may be due to an over-representation of satisfied individuals choosing to participate in the present study. The higher rate of participants scoring good to excellent global outcome compared to improvements in pain and NDI might be a result of the global perceived effect measures (such as the modified Odom) being influenced by a variety of factors and not only success from a perspective of the domains included in pain ratings and the NDI [29].

Although the 2-year radiographs showed significant differences in fusion rates between the two groups, there were no between-group differences in primary outcomes at the 20-year follow-up. These findings match those of previous follow-ups [2, 8]. Previous studies have reported differences in primary outcomes between fused and non-fused individuals regardless of group allocation [8]. Results from the present study based on how much the participants improved in self-reported disability and pain show significant differences in NDI based on fusion status, but only when adjusting for baseline values. The fusion group had slightly larger improvements in neck pain than the non-fusion group, but the differences were not statistically different. When looking at the descriptive results, it is notable that, in terms of pain, the individuals with a fused CIFC had greater pain reductions than other sub-groups. However, there were more individuals in the non-fused CIFC group, and they had a smaller reduction in pain, which might influence the mean scores of the CIFC group, and therefore no differences between surgical techniques were present. Similar rather large differences in the CIFC group based on early fusion status were noted as early as the 2-year follow-up [2].

Mean dizziness ratings were low; however balance problems and disability due to dizziness were higher, which is in line with 10-year data [30]. Mean headache intensity was notably lower at 20-year follow-up than at 10 years, and in contrast to the 10-year follow-up, no differences between surgical groups were present. Participants in the present study rated their mean health status slightly higher than at 10 years, yet lower than that of the overall Swedish population. However, a number of individuals in this study were older than average (mean age 70) and the mean population ratings decrease in older age groups [31]. Worse health was strongly correlated to neck pain and disability in the present study.

The impact of psychosocial variables was clearly demonstrated in this study by the rather high correlation coefficient between primary outcomes and self-efficacy. The major impact of sociodemographic and psychosocial variables on patient reported outcome measures such as the NDI and VAS has recently been demonstrated and underlined by Hedlund [32].

One limitation to ultra-long-term follow-ups of RCTs is the loss of participants due to death or other reasons [33]. Only 53% of the original participants were included in this 20-year follow-up. Even though the sample was rather small, 78% of participants eligible to answer the questionnaires (those not lost due to death or serious illness) did participate which may be considered a fair inclusion rate for a 20-year follow-up. Another limitation affecting the interpretation of the results are that participants may have switched treatments [33]. In the present study, participants may have had additional surgeries, employing other techniques, and/or other interventions aimed at reducing pain and disability, which may affect the long-term outcomes. Although surgical ACDF techniques have changed somewhat between 1995 and today, the procedure is basically the same. The disc is removed, the nerve roots are compressed, and the fusion is achieved by a cage or a bone graft with or without fixation with a plate. Today’s cages may allow bony ingrowth or be fixed by integrated screws, avoiding the need of plates. Overall, the techniques are biomechanically and biologically similar, with similar short/medium term outcomes. Therefore, the results of the present study are likely to reflect the outcome also of current techniques.

In conclusion, the results of this study do not support the idea that type of fusion technique affects the long-term outcome of ACDF. Pain and disability improved substantially over time, irrespective of surgical technique. However, most participants reported residual disability to a non-negligible extent. Pain and disability were correlated with lower self-efficacy and quality of life.

## Data Availability

The datasets used and/or analyzed during the current study are available from the corresponding author on reasonable request.
